# A study on the effect of different chemical routes on functionalization of MWCNTs by various groups (-COOH, -SO_3_H, -PO_3_H_2_)

**DOI:** 10.1186/1556-276X-6-583

**Published:** 2011-11-07

**Authors:** Pawan Kumar, Jin-Soo Park, Prabhsharan Randhawa, Sandeep Sharma, Mun-Sik Shin, Satpal Singh Sekhon

**Affiliations:** 1 Department of Physics, Guru Nanak Dev University, Amritsar, 143005, India; 2 Department of Environmental Engineering, College of Engineering, Sangmyung University, Cheonan, Chungnam Province, 330-720, Republic of Korea

**Keywords:** functionalization, carbon nanotubes, dispersion, surfactant

## Abstract

Pristine multiwall carbon nanotubes [MWCNTs] have been functionalized with various groups (-COOH, -SO_3_H, -PO_3_H_2_) using different single- and double-step chemical routes. Various chemical treatments were given to MWCNTs using hydrochloric, nitric, phosphoric, and sulphuric acids, followed by a microwave treatment. The effect of the various chemical treatments and the dispersion using a surfactant via ultrasonication on the functionalization of MWCNTs has been studied. The results obtained have been compared with pristine MWCNTs. Scanning electron microscopy, energy dispersive X-ray [EDX] spectroscopy, and transmission electron microscopy confirm the dispersion and functionalization of MWCNTs. Their extent of functionalization with -SO_3_H and -PO_3_H_2 _groups from the EDX spectra has been observed to be higher for the samples functionalized with a double-step chemical route and a single-step chemical route, respectively. The *I*_D_/*I*_G _ratio calculated from Raman data shows a maximum defect concentration for the sample functionalized with the single-step chemical treatment using nitric acid. The dispersion of MWCNTs with the surfactant, Triton X-100, via ultrasonication helps in their unbundling, but the extent of functionalization mainly depends on the chemical route followed for their treatment. The functionalized carbon nanotubes can be used in proton conducting membranes for fuel cells.

## Introduction

Currently, carbon nanotubes [CNTs] are the state-of-the-art materials actively studied by both experimentalists and theoreticians because of their versatile structural, electronic, mechanical, optical properties [1-3]. The pristine CNTs generally exist in bundled form due to the presence of strong Van der Waals interactions between them. In particular, these intermolecular forces of attraction are based on the pi [**π**] bond stacking phenomena between adjacent nanotubes, and there can be at least hundreds of π stacking sites between two CNTs. Hence, intermolecular forces are very strong. CNTs should be unbundled prior to their use for any application. Dispersion of nanotubes can be achieved using various surfactants, polymers, biomolecules, etc. via a physical or chemical method. In the case of surfactants, the surfactant groups get adsorbed onto the CNT surface without disturbing the π stacking system of the graphene sheet and result in dispersion. Out of the different surfactants being used for the dispersion of CNTs like sodium dodecylbenzenesulfonate [SDS], dodecyltrimethyl ammonium bromide, Tween 20 (Sigma-Aldrich, St. Louis, MO, USA), Tween 80 (ICI Americas, Inc., Wilmington, DE, USA), Triton X-100 (Dow Chemical Company, Midland, MI, USA), etc., the SDS and Triton X-100 have been reported to result in the minimum and the maximum dispersions of nanotubes, respectively [4]. Triton X-100 is mainly used to disperse CNTs due to its number of advantages including a non-covalent approach for dispersion, and the presence of a benzene ring in its chemical structure can be easily removed by washing. The most common approach is to disperse the CNTs in an aqueous surfactant solution, which is then subjected to ultrasonication in order to mechanically break the aggregation and eventually yield fully separated CNTs. The surfactant molecules are adsorbed onto the surface of CNTs (as shown in Scheme 1, see Additional file 1), have repulsion between them, and hence help to disperse the CNTs [5].

Dispersion of CNTs depends upon a number of factors, such as the type of CNTs, their geometry, the relative ratio of CNTs, and the type of surfactant being used. After dispersing the nanotubes, it is desirable to functionalize them with various chemical groups depending upon the application for which we want to use them.

Various chemical groups can be attached physically or chemically to the side walls or end caps of nanotubes, without significantly changing their desirable properties [6]. This process is called functionalization of nanotubes. A large number of methods are being used for the functionalization of CNTs, which can be broadly divided into the endohedral and exohedral methods. We have followed the exohedral mode in which the chemical groups are attached to the outer wall of the CNTs. Exohedral functionalization can be further subdivided into the covalent and non-covalent approaches. In the covalent approach, functionalization has been achieved by attaching the functional group on the side walls, end caps, or defect sites of nanotubes with a covalent bond, whereas in the non-covalent approach, chemical groups are attached by the wrapping of polymers, biomolecules, etc. on nanotubes.

In the present study, MWCNTs have been covalently functionalized with different chemical groups (-COOH, -SO_3_H, -PO_3_H_2_) using various single- and double-step chemical routes. The effect of dispersion using Triton X-100 via ultrasonication, before the functionalization of CNTs, has also been studied. The defect concentration has been determined from Raman studies. The extent of functionalization with different groups has been determined from the EDX results and chemical routes which results in the identification of sulfonation and phosphonation of higher extents.

### Experimental details

Multiwall carbon nanotubes [MWCNTs] (CNT M95, Carbon Nano-material Technology Co., Ltd., Pohang Si Nam-gu, Gyeongsangbuk-do, South Korea) with a diameter of 5 to 15 nm, a length of 10 μm, and a purity > 95% have been used as received in the present study. We have functionalized four different samples of MWCNTs. The details of these prepared samples and their codes are given in Table 1, and the methods of functionalization of each sample MWCNT are given as follows.

**Table 1:** Sample codes

**S. no**.	Amount of MWCNTs	Chemical route followed	Dispersion before functionalization	Functional groups attached	Sample code
1	40 mg	Double-step functionalization	No	-COOH -SO_3_H	FPCNT01
2	50 mg	Double-step functionalization	Yes	-COOH - SO_3_H	DFCNT03
3	50 mg	Single-step functionalization	Yes	-COOH	FCNT03
4	20 mg	Single-step functionalization	No	-COOH -PO_3_H_2_	PhCNT01

### FPCNT01

Forty milligrams of MWCNTs had been taken, and 20 mL HNO_3 _was added to it. The sample was refluxed for 240 min at 100°C. Furthermore, the sample was given multiple washings via centrifugation at 12, 000 rpm for 6 min (six times) and dried overnight in an oven at 60°C. For the second step of functionalization, a 1:1 *v*/*v *ratio of HNO_3 _and H_2_SO_4 _(15 mL each) was added to the dried sample. Microwave treatment was given for 5 min on an on/off basis. After this, 20 mL of HCl was added slowly to the sample, and it was refluxed for 60 min at an ambient temperature. In order to give the sample multiple washings, centrifugation was done at 12, 000 rpm for 6 min (six times). The functionalized sample was dried overnight in an oven at 60°C.

### FCNT03

Fifty milligrams of MWCNTs had been taken and dispersed with 1.9% Triton X-100 and 200 mL of deionized [DI] water via ultrasonication for 120 min. After this, the sample had been given multiple washings through centrifugation at 7, 000 rpm for 10 min (six times) and dried overnight in an oven at 60°C. For the functionalization, a 1:1 *v*/*v *ratio of HNO_3 _and HCl (25 mL each) was added to the dried dispersed sample, and it was refluxed for 90 min at 80°C and then centrifuged at 12, 000 rpm for 10 min (six times). The functionalized sample was dried overnight in an oven at 60°C.

### DFCNT03

Fifty milligrams of MWCNTs had been taken and dispersed with 1% Triton X-100 and 200 mL DI water via ultrasonication for 60 min. After this, the sample had been given multiple washings through centrifugation at 12, 000 rpm for 6 min (six times) and dried overnight in an oven at 60°C. Furthermore, a 1:1 *v*/*v *ratio of HNO_3 _and HCl (25 mL each) was added to the dried dispersed sample, and it was refluxed at 80°C for 90 min. The sample had been given multiple washings via centrifugation at 12, 000 rpm for 6 min (six times) and dried overnight in an oven at 60°C. For the second step of functionalization, a 1:1 *v*/*v *ratio of HNO_3 _and H_2_SO_4 _(25 mL each) was added to the dried sample, and microwave treatment was given for 5 min on an on/off basis. After this, 30 mL HCl was added slowly to the above mixture. The sample was then refluxed for 60 min at an ambient temperature, followed by centrifugation at 12, 000 rpm for 6 min (six times). The sample was dried overnight in an oven at 60°C.

### PhCNT01

Twenty milligrams of MWCNTs had been taken, and 10 mL of H_3_PO_4 _was preheated at 60°C for 20 min and then added to the CNTs. Furthermore, 10 mL of HNO_3 _was added to the above mixture. It was mixed and refluxed at 130°C for 60 min. In order to give multiple washings, the sample was centrifuged at 12, 000 rpm for 6 min (six times) and dried overnight in an oven at 60°C.

### Transmission electron microscopy

Transmission electron microscopy [TEM] (Libra 120, Carl Zeiss AG, Oberkochen, Germany) at an acceleration voltage of 120 kV was used to examine the size and distribution of the CNT surface of various samples. The TEM specimens were prepared by placing a few drops of the sample solution on a lacey carbon grid.

### Scanning electron microscopy

Scanning electron microscopy [SEM] micrographs were obtained with a Hitachi S-4800 field-emission SEM (Hitachi High-Tech, Minato-ku, Tokyo, Japan) at an acceleration voltage of 0.5 to 30 kV. Specimens for high-resolution imaging were coated with Osmium.

### Energy dispersive X-ray

The energy dispersive X-ray [EDX] (X-Max 50011, HORIBA Ltd., Minami-Ku, Kyoto, Japan) spectra were obtained to determine the elemental information on the CNT at 16 kV and 15 μA.

### Raman spectra

Raman spectroscopy was carried out at room temperature using a FRA 106/S (BRUKER OPTIK GMBH, Ettlingen, Germany) Raman spectrometer, with a 1006-nm Nd-YAG laser and a 4-cm^-1 ^resolution.

## Results and discussion

Pristine CNTs are generally chemically inert and insoluble in many solvents. In order to make them suitable for various applications, they have to be functionalized with different groups. The functionalized CNTs are soluble in various organic solvents. The functionalization of CNTs strongly depends upon the chemical route followed. In the present study, different chemical routes have been used for the functionalization of CNTs with the -COOH, -SO_3_H, and -PO_3_H_2 _groups, and their effects on the functionalization have been studied. MWCNTs have been functionalized with the -COOH, -SO_3_H, and -PO_3_H_2 _groups using various chemical routes given in Scheme 2 (see Additional file 2):

- Single-step process (FCNT03 and PhCNT01)

- Double-step process (FPCNT01 and DFCNT03)

- Without dispersion with surfactant (FPCNT01 and PhCNT01)

- After dispersion with surfactant (DFCNT03 and FCNT03).

**Figure 1:**
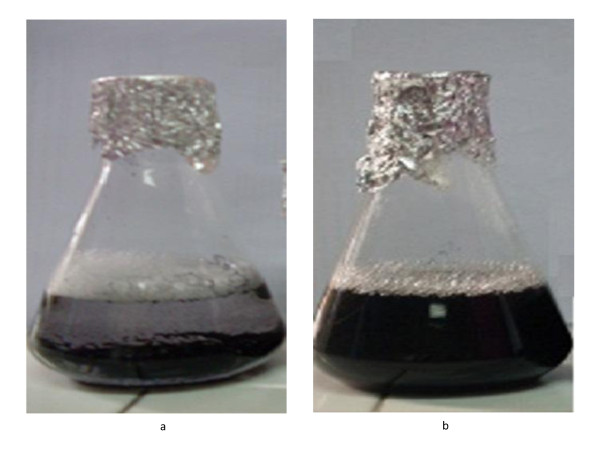
**Sample photographs before (a) and after (b) ultrasonication (photograph taken after 24 h of dispersion)**.

**Figure 2:**
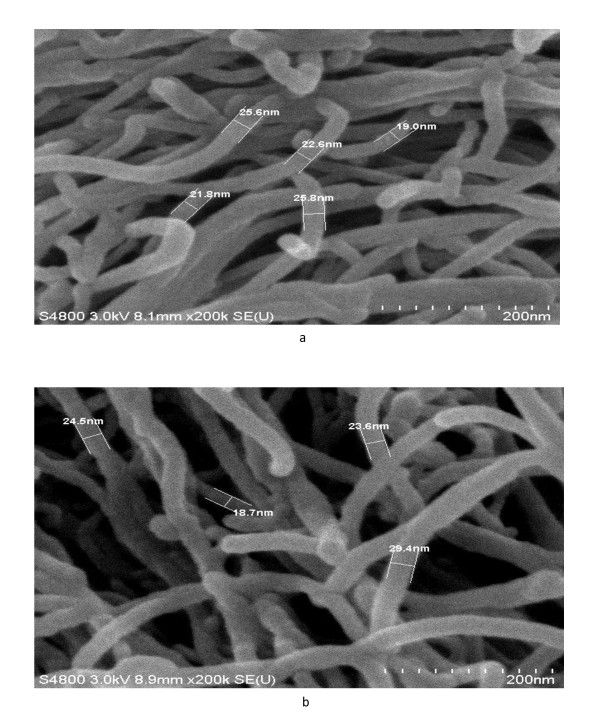
**SEM micrographs of CNTs before (a) and after (b) dispersion**.

The photographs of MWCNTs before and after sonication are given in Figure 1. Pristine CNTs are not soluble in water and settle down at the bottom of the flask as observed in Figure 1. However, after sonication for one hour, CNTs are dispersed, and a uniform solution is obtained as observed in Figure 1. The dispersion of CNTs after sonication was also studied by SEM. The SEM micrographs of CNT samples before and after sonication are given in Figure 2. The SEM micrograph of pristine CNTs shows the presence of bundles and ropes of nanotubes, which have been observed to be dispersed after sonication.

**Figure 3:**
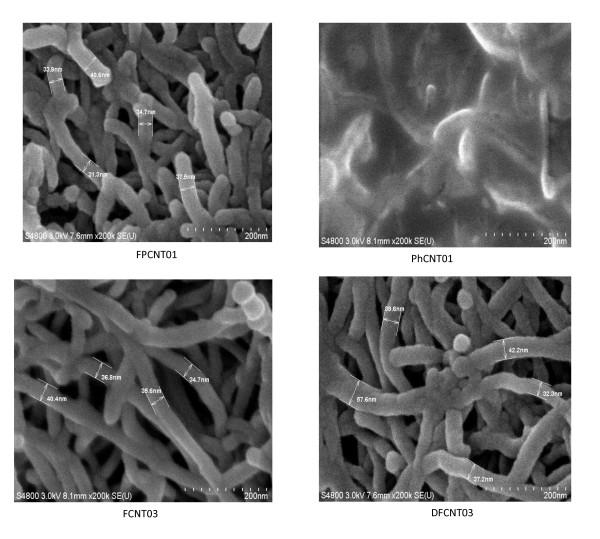
**SEM micrographs of different functionalized samples**.

**Figure 4:**
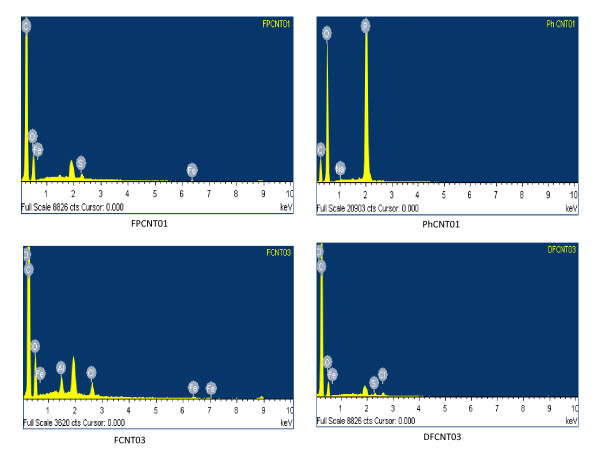
**EDX spectra for different samples**.

The functionalization of MWCNTs with different groups using single- and double-step chemical routes has also been studied by SEM, and the micrographs for different samples are given in Figure 3. The bundles present in the pristine sample have been also observed to be dispersed after the functionalization of MWCNTs by different groups. Samples FPCNT01 and PhCNT01, which have been functionalized after sonication, show dispersion which takes place due to sonication as well as functionalization. The extent of dispersion is better in the sample, PhCNT01, which has been functionalized with the -COOH and -PO_3_H_2 _groups. The SEM micrographs also confirm the presence of the attached groups on the outer walls of MWCNTs. The presence of the different chemical groups (-COOH, -SO_3_H, and -PO_3_H_2_) on the walls of the MWCNTs and their quantitative amounts have also been studied by EDX. The EDX plots for the different samples are given in Figure 4, which shows the presence of carbon, oxygen, sulfur, and phosphorus in the functionalized samples. Since the as-received MWCNTs used in the present study are 95% pure, some catalytic elements are also present in small amounts and are detected in the EDX results. The quantitative (weight and atomic percent) amounts of the different elements (C, O, S, P) present in these samples have been calculated from the EDX data, and their values are listed in Table 2. From the EDX data, it has been observed that out of the two samples, FCNT03 and PhCNT01, which have been functionalized by a single-step chemical route, sample PhCNT01 is better functionalized (phosphorus content 25 wt.%). The SEM results for this sample (Figure 3) also confirm its better functionalization. This shows that the use of H_3_PO_4 _acid for functionalizing CNTs is the most effective, and a large number of -PO_3_H_2 _groups are attached. For samples FPCNT01 and DFCNT03, which have been functionalized with the -COOH and -SO_3_H groups using a double-step chemical route, the EDX data show that the functionalization with the -SO_3_H group is better in sample FPCNT01 than in DFCNT03. The sulfur content in FPCNT01 and in FPCNT03 is 0.6 and 0.34 wt.%, respectively. This shows that the double-step chemical route followed for the functionalization of sample FPCNT01 is relatively more effective for the sulfonation (-SO_3_H) of MWCNTs, whereas the maximum phosphonation (-PO_3_H_2_) has been achieved for sample PhCNT01 which was functionalized with a single-step chemical route. It shows that, ultimately, the more important step for functionalization of MWCNTs is the chemical route followed for their treatment even though dispersion assists in the unbundling of CNTs. Functionalization also assists in the dispersion of MWCNTs.

**Table 2:** Concentration of different elements from EDX data

Element	C		O		P		S	
Sample	(w.%)	(at.%)	(w.%)	(at.%)	(w.%)	(at.%)	(w.%)	(at.%)
FPCNT01	82	87	16	14	-	-	0.60	0.24
PhCNT01	24	34	50	52	25	13	-	-
FCNT03	78	83	18	15	-	-	-	-
DFCNT03	86	89	13	10	-	-	0.34	0.13

**Figure 5:**
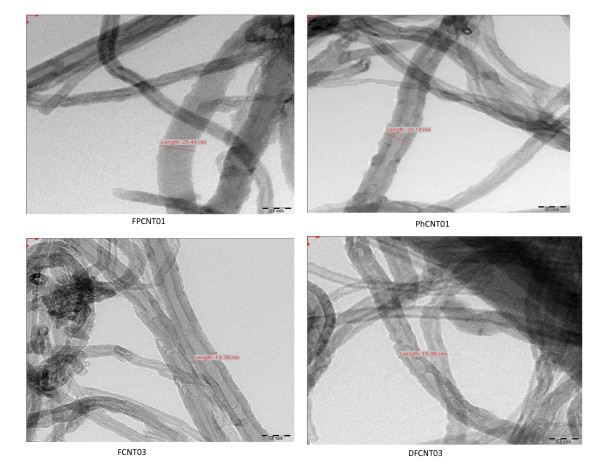
**TEM images for different samples**.

The dispersion and functionalization of MWCNTs with various groups were also confirmed by TEM studies, and the TEM micrographs of the different samples are given in Figure 5. Sample FPCNT01, which has been functionalized with the -COOH and -SO_3_H groups, shows dispersion. Sample PhCNT01, which has been functionalized with -PO_3_H_2 _groups, shows a larger functionalization which is also supported by the EDX results (Table 2). The TEM micrographs show that sample FPCNT01, which shows a relatively higher degree of sulfonation (Table 2), also shows better dispersion as compared with sample DFCNT03. Thus, for sulfonation of CNTs, the chemical route followed for the functionalization of sample FPCNT01 shows better results.

**Figure 6:**
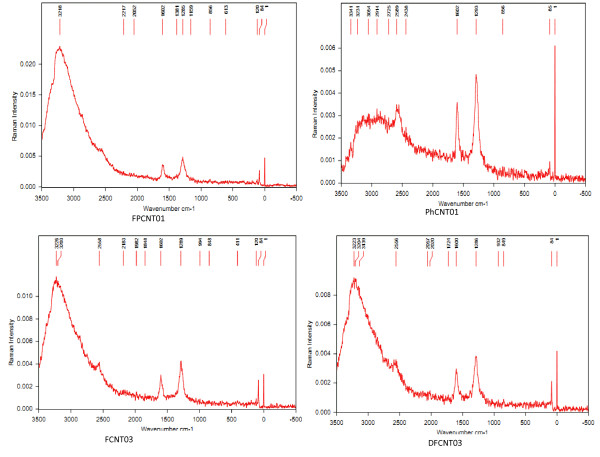
**Raman spectra for different samples**.

The chemical route followed for the functionalization of CNTs, as given in Scheme 1, involves the use of strong acids (HCl, HNO_3_, H_2_SO_4_, and H_3_PO_4_). These acids have been reported to damage the surface of CNTs and also create defects which generally act as potential sites for the attachment of different chemical groups. The defect concentration in CNTs can be studied by Raman spectroscopy. The Raman spectra of the different samples have been recorded and are shown in Figure 6. The most intense band near 1, 600 cm^-1 ^is the characteristic band (G band) of graphene and is due to the in-plane vibrations of carbon atoms. The band near 1, 280 cm^-1 ^is due to the disorder or structural defects (D band) in the graphene sheet. The ratio of the intensities of the D and G bands (*I*_D_/*I*_G_) is generally taken as a measure of the defect concentration. This ratio has been calculated for the different samples from the Raman data, and the values are listed in Table 3. The ratio is highest for sample FCNT03, which shows that the chemical route followed for the functionalization of this sample creates a larger number of defects on the surface of CNTs. Similarly, out of samples FCNT03 and DFCNT03, this ratio is higher for sample FCNT03 which also shows a higher degree of functionalization as observed from EDX results. Thus, it has been observed that the chemical route followed for the functionalization of CNTs plays an important role and must be optimized for proper functionalization.

**Table 3:** Intensities of the G and D bands and intensity ratio (*I*_D_/*I*_G_) calculated from Raman data

Sample	G band		D band		***I*_D_/*I***_**G**_
	Position of peak (cm^-1^)	Intensity	Position of peak (cm^-1^)	Intensity	
**FPCNT01**	1602	0.00363	1285	0.00484	1.3333
**PhCNT01**	1602	0.00359	1293	0.00483	1.3454
**FCNT03**	1602	0.00304	1289	0.00428	1.40789
**DFCNT03**	1600	0.00296	1286	0.00383	1.3454

## Conclusions

MWCNTs have been functionalized with different groups using various single- and double-step chemical routes. The maximum sulfonation (functionalization with -SO_3_H groups) has been achieved for sample FPCNT01 which was functionalized using a double-step chemical route, whereas the maximum phosphonation (functionalization with -PO_3_H_2 _groups) has been achieved for sample PhCNT01. The highest defect concentration (I_D_/I_G_) has been observed for sample FCNT03, which has been functionalized with a single-step process using HNO_3_. The dispersion of CNTs using a surfactant helps in their unbundling, but the more important step is the chemical route followed for their functionalization as observed from EDX results. A proper choice of the chemical route and the amount of acid used can be helpful to control the extent of functionalization with various chemical groups. The incorporation of CNTs functionalized with the -SO_3_H and -PO_3_H_2 _groups in sulfonated polymers can be used as high temperature fuel cell membranes.

## Competing interests

The authors declare that they have no competing interests.

## Authors' contributions

PR, PK, and SS prepared the samples. JSS and MSS helped in the characterization studies. SSS and PR conceived the study and participated in the study and analysis. All authors contributed equally and also approved the final manuscript.

## Supplementary Material

Additional file 1 **Scheme 1**. Mechanism of dispersion of CNTs [5]. Click here for file

Additional file 2 **Scheme 2**. Chemical route followed for the functionalization of different samples. Click here for file
