# Supplementary material for: A study on the effect of different chemical routes on functionalization of MWCNTs by various groups (-COOH, -SO3H, -PO3H2)
Source: Nanoscale Res Lett. 2011 Nov 7;6(1):583. doi: 10.1186/1556-276X-6-583 (PMC3220730; doi:10.1186/1556-276X-6-583)
Supplement: Additional file 1 — Scheme 1. Mechanism of dispersion of CNTs [5]. [file 1556-276X-6-583-S1.DOCX]

Scheme 1
